# Clinical analysis of selected complement-derived molecules in human adipose tissue

**DOI:** 10.1186/1479-5876-11-11

**Published:** 2013-01-09

**Authors:** Wojciech Błogowski, Marta Budkowska, Daria Sałata, Karol Serwin, Barbara Dołęgowska, Marek Łokaj, Piotr Prowans, Teresa Starzyńska

**Affiliations:** 1 Department of Gastroenterology, Pomeranian Medical University in Szczecin, ul. Unii Lubelskiej 1, 71-252, Szczecin, Poland; 2 Department of Laboratory Diagnostics and Molecular Medicine, Pomeranian Medical University in Szczecin, Szczecin, Poland; 3 Department of Physiology, Pomeranian Medical University in Szczecin, Szczecin, Poland; 4 Department of Plastic, Endocrine and General Surgery, Pomeranian Medical University in Szczecin, Szczecin, Poland

**Keywords:** Adipose tissue, Complement cascade, Obesity

## Abstract

**Background:**

It has been suggested that action of complement cascade [CC]-derived anaphylatoxins/molecules may represent a missing link between obesity and metabolic disorders. However, to date, the direct biochemical/immunomodulatory composition of the human AT environment remains poorly understood. In this study, we examined plasma and AT (subcutaneous and visceral/omental) levels of selected CC-derived anaphylatoxins/molecules, and adipsin as well as verified their associations with immune and stem cells chemoattractant - stromal-derived factor-1 (SDF-1).

**Methods:**

A total of 70 (35 subcutaneous and 35 omental) AT samples were obtained from patients undergoing elective surgery. Plasma and AT-derived interstitial fluid levels of C3a, C5a, C5b-9/membrane attack complex (MAC), complement factor D (adipsin) were measured using ELISA.

**Results:**

AT levels of all examined substances were significantly lower than the corresponding levels in the plasma (in all cases P < 0.0000001). Moreover, in subcutaneous AT, robust C3a and adipsin concentrations were observed, whereas high levels of C5b-9/MAC were detected in the visceral depots. In addition, we established the correlations between analyzed molecular substances and body composition, BMI and/or the adiposity index of the examined patients.

**Conclusions:**

Our study demonstrated for the first time that significantly reduced levels of complement-derived molecules were present in human AT than in the peripheral blood, and that these factors are associated with the metabolic status of examined individuals. Moreover, in human AT, various associations between complement-derived molecules and SDF-1 levels exist.

## Background

Human adipose tissue (AT) is a very dynamic organ that profoundly contributes to the regulation of several (patho-) physiological processes, including embryonic development, systemic endocrine/metabolic homeostasis, and immunomodulation. This orchestration by AT on such diverse processes may be mediated by its molecular properties. For example, AT synthesizes and/or secretes numerous hormones, cytokines, and growth factors into peripheral blood, which act on an autocrine, paracrine, and/or peripheral level to influence systemic homeostasis in humans [1-4].

From the clinical standpoint, accumulation of visceral/omental AT (central obesity) is strongly associated with presence of several metabolic abnormalities, such as glucose intolerance, hyperglycemia, hypertriglyceridemia and/or other features of “metabolic syndrome”. The exact mechanisms responsible for this link are neither fully examined nor understood. However, it has been suggested that action of various compounds of the innate immune system (mainly complement cascade [CC]-derived anaphylatoxins/molecules) may represent a missing link between obesity and metabolic disorders [5-8]. Indeed, various genetic studies have shown that animal and human AT express CC genes in different depots, and this is strongly related to the systemic metabolic status. In addition, AT is believed to be an important source of selected CC-derived anaphylatoxins/molecules and adipsin, which genetic expression seems to differ between subcutaneous and visceral/omental depots [9-12]. Unfortunately, several translational issues still remain to be clarified; these include need for verification/analysis of: (i) the exact biochemical concentrations of complement-derived substances in the human AT environment, (ii) their relation to the systemic/peripheral levels, and (iii) their potential associations with the human metabolic status/body composition.

In addition, in recent years it has become evident that AT environment may also offer potential niches for bone marrow (BM)-derived immune and/or (hematopoietic) stem/progenitor cells (SCs) [13]. This phenomenon is believed to be also supported by potential action of selected complement cascade (CC)-derived anaphylatoxins and molecules [14]. Namely, they may act as chemoattractants, stimulate the release of powerful bioactive lipids and enhance homing signals of stromal-derived factor-1 (SDF-1) and its receptor (CXCR4) that are essential for the successful directing and anchoring of immune and stem cells to the target tissues [15,16]. In our previous study, we have demonstrated that SDF-1 levels in human AT are significantly lower than in plasma; higher levels of this chemoattractant are observed in visceral/omental AT, as well as, these are associated with several parameters describing human metabolic status [17]. Currently, there is no data indicating, whether in human AT any associations between levels of the CC-derived substances and SDF-1 occur. Therefore, we addressed all of these issues by comprehensively analyzing the levels of the terminal panel of CC-derived molecules (C3a, C5a, and C5b-9), and complement factor D (adipsin) in human subcutaneous and visceral/omental AT. Furthermore, we verified how the levels of these substances differed between lean, overweight, and obese subjects, as well as, their associations with previously reported SDF-1 values. Our hypothesis was that different depots of human AT possess significantly different levels of CC-derived molecules and these molecules are associated with the metabolic status/body composition of an individual and SDF-1 levels.

## Material and methods

### Patients and medical procedure

We recruited 35 healthy patients who underwent elective surgery at the Department of Plastic, Endocrine and General Surgery of the Pomeranian Medical University in Szczecin between January 2011 and January 2012. Patients with signs of active inflammation, autoimmune systemic diseases, liver dysfunction, and/or current hormonal abnormalities were excluded from the study. None of the patients were under hormonal medication, had significantly gained/lost weight in the 3 months before surgery, or had evidence of metabolic diseases other than obesity. Liver disease and thyroid dysfunction were specifically excluded by biochemical workup. All patients underwent detailed clinical phenotyping in a fasted state (12 h, overnight). Their body weight was measured in light clothing and approximated up to the nearest 0.1 kg; their height was measured using a stadiometer with the subjects standing barefoot. Further, the subjects’ body mass and adiposity indexes (BMI and BAI, respectively) were calculated according to standard formulas [18]. Waist circumference (expressed in cm) was measured at the narrowest point between the lowest rib and the iliac crest, and the waist-to-hip ratio (WHR) was calculated. Blood pressure was measured in the supine position on the right arm after rest (for at least 15 min). A standard sphygmomanometer of appropriate cuff size was used, and the first and fifth phases were recorded. Values used in this analysis were the average of 3 readings taken at 5-min intervals. In addition, all the patients underwent general biochemical blood tests. They were classified according to their BMI values as lean (19–25 kg/m^2^), overweight (25–30 kg/m^2^), or obese (≥30 kg/m^2^). General characteristics of the study subjects, together with a statistical comparison of their features between the analyzed groups, are presented in Table 1.

**Table 1:** **General characteristics of individuals enrolled in the study** (**mean** ± **standard deviation**)

**PARAMETER/GROUP**	**Healthy(n** = **11)**	**Overweight(n** = **12)**	**Obese(n** = **12)**
Age (years)	46 ± 14	50 ± 6	50 ± 16
Gender (M-male/F-female)	3-M/8-F	3-M/9-F	2-M/10-F
Height (cm)	164 ± 11	165 ± 4	167 ± 8
Weight (kg)	55 ± 5	75 ± 6*	93 ± 14*#
BMI (kg/m^2^)	20.41 ± 1.80	27.93 ± 1.70*	33.42 ± 3.20*##
BAI	19.75 ± 8.22	32.00 ± 3.16**	35.00 ± 9.38***
Waist circumference (cm)	69.20 ± 3.42	90.60 ± 9.79**	105.17 ± 4.88**
Hip circumference (cm)	80.75 ± 10.94	105.80 ± 3.49**	112.5 ± 16.90**
WHR	0.85 ± 0.11	0.86 ± 0.08	0.95 ± 0.11
Systolic blood pressure (mmHg)	135.33 ± 9.20	131.43 ± 12.84	128.50 ± 13.37
Diastolic blood pressure (mmHg)	78.33 ± 7.87	82.86 ± 9.92	80.13 ± 8.32
MAP (mmHg)	97.14 ± 6.54	98.89 ± 10.62	96.09 ± 9.22
C-reactive protein (mg/L)	2.19 ± 0.37	2.36 ± 0.95	2.31 ± 0.52
Presence of hypertension	3	3	3
Smokers	2	2	1

BMI – body mass index; BAI – body adiposity index; WHR – waist/hip ratio; MAP – mean arterial pressure.

*P < 0.001; **P < 0.01; ***P < 0.05 (vs healthy); #P < 0.05; ##P < 0.005 (vs overweight).

AT samples were collected from subcutaneous and omental/visceral depots during elective surgical procedures (laparoscopic cholecystectomy). Both AT samples were obtained from the abdomen using sterile surgical equipment, without diathermy/coagulation and with particular attention to avoid contamination with blood. The subcutaneous samples were taken from the upper midline of the abdomen, halfway between the xiphoid process and the umbilicus. The intra-abdominal AT samples were collected from the major omentum in the upper right quadrant of the abdominal cavity, in close proximity to the front wall of the liver. AT samples were excised at the beginning of the operations, placed on ice, and transported to the laboratory. The time between the excision of an AT sample, its evacuation from the abdominal walls, and its placement on ice was consistent and never longer than 35 s.

### Biochemical assays of complement-derived substances and adipsin in plasma and fat interstitial fluid (FIF)

Blood samples were collected from every patient in a fasted state (12 h, overnight), and these samples were immediately centrifuged (10 min, 1000 × *g*, 4°C). The plasma obtained was transferred to a fresh tube and stored at −80°C until further assessment.

In order to extract FIF, AT samples were processed according to previously described methods and protocols [4,17,19,20]. Plasma and AT-derived FIF concentrations of complement-derived anaphylatoxins and molecules (C3a, C5a, and C5b-9/MAC) as well as those of adipsin were measured using commercially available ELISA kits (distributed by *BD Bioscience* and *R*&*D Systems*) according to the manufacturers’ instructions.

### Statistical analysis

For comparison of the mean parameter values between the examined groups Mann–Whitney test was performed. Differences between concentrations of analyzed parameters in different AT depots, as well as, between plasma and selected AT fraction were assessed by Wilocoxon’s paired test. The strength of correlations between examined clinical and molecular parameters was calculated using Spearman’s rank test. Statistical analysis was performed using SPSS statistical analysis software. Statistical significance was defined when p values were less than 0.05.

This study protocol was approved by the Bioethical Committee of the Pomeranian Medical University in Szczecin, and patients provided informed written consent for participation after the purpose of the study was explained to them.

## Results

In our study significant differences were found between lean individuals and overweight/obese patients in terms of weight, BMI, BAI, as well as, waist and hip circumference values (Table 1). Moreover, our obese patients had significantly higher values of weight and BMI parameters than subjects classified to overweight group.

### Plasma and AT levels of the terminal panel of complement anaphylatoxins and molecules (C3a, C5a, C5b-9/MAC)

**Figure 1:**
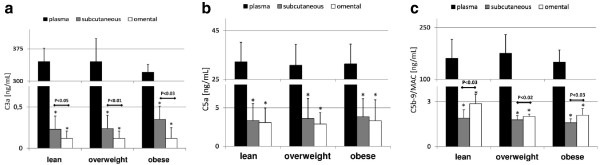
**Mean values of plasma and adipose tissue levels of complement C3a (a), C5a (b) and C5b-9/MAC (c) in healthy, overweight and obese individuals, and their statistical comparison between examined groups.** * p < 0.0000001 (vs plasma level in appropriate group).

Complement C3a, C5a, and C5b-9/MAC levels are depicted on Figure 1. Our study demonstrated that no statistically significant differences occur in terms of plasma complement C3a, C5a and C5b-9/MAC levels between lean, overweight and obese individuals. However, there was a tendency towards decrease in systemic C3a levels in obese patients (P = 0.09 in comparison to healthy and overweight group). Nevertheless, AT concentrations of C3a were significantly lower than those circulating in peripheral blood (P < 0.0000001 in all cases), and were around 2-times higher in subcutaneous than visceral depots in all examined groups (Figure 1a). In comparison to lean and overweight subjects, significantly higher levels of this complement anaphylatoxin were found in subcutaneous fraction of adipose tissue derived from obese individuals (P < 0.05 for both). Interestingly, we have observed a set of significant correlations between subcutaneous C3a levels and clinical features of examined patients, such as BMI (r = 0.31, P < 0.05), waist and hip circumference (r = 0.44 and r = 0.47, P < 0.05 respectively).

Relatively analogical results were found in terms of complement C5a analysis (Figure 1b). Namely, AT levels of this anaphylatoxin were significantly lower than those observed in peripheral blood; however, in all groups similar concentrations were observed in both – subcutaneous and visceral adipose depots. Interestingly, in our study we have observed a negative correlation between systemic C5a levels and WHR parameter (r = −0.49, P < 0.04), as well as, visceral/omental C5a concentrations were correlating with such parameters as age (r = −0.68, P < 0.002) or height (r = 0.43, P < 0.04).

Interestingly, although AT-derived FIF levels of C5b-9/MAC were also found to be significantly lower than in plasma, statistically higher concentrations of this complement molecule were present in omental fraction than in subcutaneous depots (Figure 1c). In addition, overweigh and obese subjects had significantly lower visceral/omental C5b-9 concentrations in comparison to lean individuals (P < 0.005 and P < 0.03, respectively), however, its subcutaneous levels were comparable between all examined groups. In our study, visceral/omental C5b-9 levels were strongly correlated with such clinical features as BMI (r = −0.58, P < 0.004), waist and hip circumference (r = −0.65, P < 0.005 and r = −0.47, P < 0.05, respectively), as well as, MAP values (r = −0.38, P < 0.05).

### Complement factor D/adipsin levels

**Figure 2:**
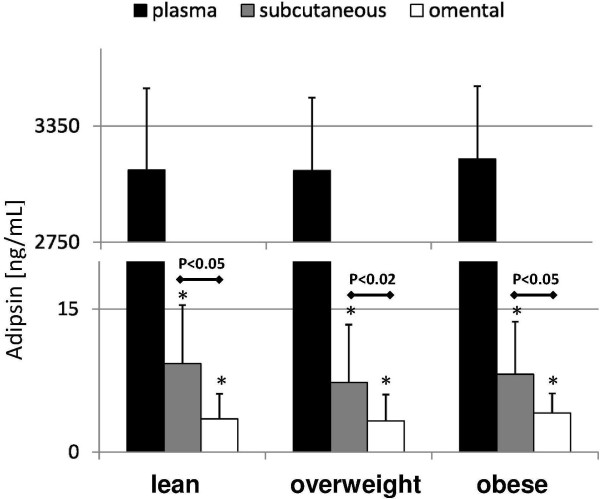
**Mean values of plasma and adipose tissue levels of factor D/adipsin in healthy, overweight and obese individuals, and their statistical comparison between examined groups.** *p < 0.0000001 (vs plasma level in appropriate group)

Analogically to the results obtained during analysis of the terminal panel of complement cascade anaphylatoxins/molecules, also plasma adipsin levels were found to be comparable between examined groups of patients, and its concentrations detected in both AT depots were significantly lower than in peripheral blood (Figure 2). Interestingly, significantly higher adipsin levels were found in subcutaneous fraction of AT, and these were comparable between all groups of analyzed patients. Plasma adipsin concentrations were correlated with patients’ age (r = −0.47, P < 0.03), BAI (r = −0.63, P < 0.005) and WHR values (r = 0.55, P < 0.02). Adipose tissue levels of complement factor D were also associated with plasma C3a concentrations (r = 0.38, P < 0.05 for both).

### CC-derived anaphylatoxins/molecules and chemoattractant for immune/stem cells – SDF-1

Finally, we verified whether observed systemic and/or AT levels of examined CC-derived anaphylatoxin/molecules are associated with levels of chemoattractant for bone marrow-derived cells, such as SDF-1 (that we reported previously by us [17]). Results of our analyses demonstrated that AT SDF-1 levels were inversely associated with C5b-9/MAC levels in both – subcutaneous (r = −0.31) and visceral (r = −0.30) fractions (P < 0.05 for both). No other significant associations between examined CC-derived substances and SDF-1 were observed.

## Discussion

In recent years, much effort has been put into discovering the diverse roles of CC anaphylatoxins/molecules. Their actions appear to be crucial for the regulation of several processes that extend beyond immunomodulation. CC influence in successful human (embryonic/prenatal) development, organ regeneration, and/or SC homing in various tissues/organs has been especially highlighted in some previous reports [15,16,21-24]. Therefore, we examined the biochemical-immunological composition of the human AT environment. To this end, we performed a comprehensive analysis of AT levels of the terminal panel of complement-derived anaphylatoxins/molecules and those of the complement factor D/adipsin. Furthermore, we verified their potential association with SDF-1, which is a well-known chemoattractant for immune and (hematopoietic) stem cells.

In our study, we found that the levels of all examined factors in the AT were drastically lower than in the peripheral blood. In particular, C3a and adipsin levels in both AT depots were approximately 250- to 1000-fold lower than their corresponding concentrations in plasma. In contrast, relatively high C5a levels were detected in both AT fractions, and these levels were found to be only 10-fold lower than those detected in the peripheral blood. Moreover, levels of only a select few of these substances significantly differed between patients with varying status of weight gain; however, these variations were in relatively narrow ranges. These results indicate that while the biochemical composition of the human AT environment seems to be affected by changes in the metabolic profile of the individual, a relatively comparable constellation of immunomodulatory compounds exist within its environment.

In addition, our results showed depot-specific differences in AT-derived FIF levels of the examined complement anaphylatoxins/molecules. We found that approximately 2-fold higher levels of complement C3a and adipsin are present in the subcutaneous fraction of human AT, whereas visceral/omental depots possessed higher levels of C5b9-MAC. Several authors previously showed that AT depot-specific changes may occur in the genetic expression of CC-derived substances in animals or humans, and these changes are associated with as well as may vary according to the metabolic status of an individual [7,11,12]. The results of our study indirectly support these previously reported findings because we observed several significant differences in the levels of CC-derived substances between subcutaneous and visceral/omental fractions of AT, and, to a certain extent, these differences were associated with parameters such as BMI, BAI, and/or WHR values. Nevertheless, the precise nature of these associations remains to be elucidated in further laboratory and/or clinical studies.

We would also like to highlight our observation that in human AT strong correlations between selected complement-derived molecules (mainly C5b-9/MAC) and SDF-1 are observed. This cross-talk between C5b-9/MAC and SDF-1 signaling is already known to be very important for successful mobilization of various SCs and lymphocytes from BM and proper homing to target tissues [15,16]. Whereas the exact molecular mechanisms of this association have not been discovered, results from recent experimental studies suggest an indirect interaction between these two molecules. For example, C5b-9/MAC has been proved to activate intracellular MAP ^p44/42^ and AKT signaling in murine hematopoietic SCs, and even though the action of MAC does not upregulate the expression of the CXCR4 receptor on BM-derived SCs, it may stimulate SDF-1 release in the BM stroma and increase adhesiveness of SCs to “niches” in the target homing tissue [25]. We speculate that because of the fact that SDF-1 levels are relatively high in human AT and are strongly associated with selected complement-derived molecules, it is possible that actions of these molecules may influence and promote the homing of immune cells and hematopoietic SCs in human AT, and thereby contribute to development of “obesity-associated” inflammation within AT environment. Nevertheless, this phenomenon requires further investigations.

Finally, a recent study showed that various complement-derived substances can also significantly influence AT-derived SC homeostasis in experimental *in vitro* culture conditions [26]. To our knowledge, our study is the first one on human AT that delivers explicit information about the direct (changes in the) estimated “ranges” of biochemical levels of complement-derived substances in lean and overweight/obese subjects. Thus, our results undoubtedly assist other principal investigators allowing them to adjust their experimental approaches to mimic real (patho-) physiological conditions that occur in human AT. However, it is important to highlight that those can be slightly different from the exact CC concentrations present in AT environment due to potential “release” of complement molecules from lysed adipocytes during technical processing of AT samples in our study.

## Conclusions

In summary, our study shows that (i) within the human AT environment lower levels of complement-derived anaphylatoxins/molecules and adipsin are present than the levels in the peripheral blood, (ii) differences in the concentrations of these factors are found between fat depots, and (iii) these substances appear to be associated with the metabolic status/body composition of an individual. Finally, our study highlights that complement-derived molecules and SDF-1 are associated in various ways in human AT. Further clinical studies are necessary to verify their role in the regulation of various hematopoietic cells homeostasis and homing in human AT.

## Abbreviations

AT: Adipose tissue; BAI: Body adiposity index; BM: Bone marrow; BMI: Body mass index; CC: Complement cascade; FIF: Fat interstitial fluid; MAC: Membrane attack complex; SC(s): Stem cell(s); SDF-1: Stromal-derived factor-1.

## Competing interests

The authors declare that they have no competing interests.

## Authors’ contributions

WB: designed the study, collected clinical data, performed statistical analysis, wrote and revised the manuscript; MB, DS, KS, BD: adapted biochemical protocols and performed biochemical analyses; MŁ, PP: operated included patients and collected perioperatively adipose tissue samples; TS: revised the manuscript drafts and helped in creation of a final version of the article. All authors have read and approved the final manuscript.
